# Evaluating the Effectiveness of Stenting for Aortic Coarctation

**DOI:** 10.1055/s-0042-1750097

**Published:** 2022-12-20

**Authors:** Constantinos Contrafouris, Constantine N. Antonopoulos, Spyridon Rammos, Meletios Kanakis, Konstantinos Petsios, John D. Kakisis, George Geroulakos

**Affiliations:** 1 1st Cardiac Surgery Department, “Onassis” Cardiac Surgery Center, Athens, Greece; 2 Department of Vascular Surgery, Medical School, National and Kapodistrian University of Athens, 12462 Athens, Greece; 3 Department of Pediatric Cardiology, “Onassis” Cardiac Surgery Center, Athens, Greece; 4 Department of Pediatric and Congenital Heart Surgery, “Onassis” Cardiac Surgery Center, Athens, Greece; 5 Nursing Clinical Research Office, “Onassis” Cardiac Surgery Center, Athens, Greece

**Keywords:** aortic coarctation, endovascular, stent, covered, uncovered

## Abstract

**Background** Coarctation of the aorta (CoA) is a congenital cardiovascular malformation involving narrowing of the thoracic aorta just distal to the left subclavian artery. The aim of our study was to evaluate the hemodynamic effects of endovascular treatment for CoA by using invasive aortic catheterization.

**Methods** All patients with CoA who underwent treatment by aortic stent implantation between September 1, 2003, and February 1, 2019, at the “Onassis Cardiac Surgery Center,” in Athens, Greece, were evaluated. Patients were treated with either bare (uncovered) Cheatham-Platinum (bCP) or covered Cheatham-Platinum (cCP) stent implantations. Invasive aortic pressure measurements were recorded before and after the endovascular intervention.

**Results** A total of 48, eight zig CP stents, comprising 24 bCP and 24 cCP stents were implanted in 47 patients. The mean aortic diameter (mm) at the CoA lesion increased from 9.7 ± 3.3 to 19.2 ± 2.9 mm ( *p* <0.01) after the endovascular procedure. The invasive mean blood pressure (BP; mm Hg) from catheterization in the descending aorta increased (before = 114.2 ± 12.8 vs. after = 135.5 ± 28.1; *p* <0.01), while the invasive mean BP (mm Hg) from catheterization in the ascending aorta was decreased (before = 156.8 ± 25.0 vs. after = 138.4 ± 27.5; *p* <0.01) after the intervention. The mean aortic BP gradient decreased in both types of stents after intervention (BP gradient among patients with cCP stents = 30.9 +/− 23.6 mmHg and BP gradient among patients with bCP stents = 38.0 +/−23.1 mmHg). However, there was no statistically significant difference between the two types of stents; *p* = 0.36.

**Conclusions** Invasive aortic catheterization provided evidence that endovascular stenting with either bare or covered stents is efficient in treating patients with CoA.

## Introduction

Coarctation of the aorta (CoA) is a congenital cardiovascular malformation mainly referring to narrowing of the thoracic aorta just distal to the left subclavian artery. 1 CoA is normally detected and repaired surgically in childhood, but it occasionally recurs in adolescence or adulthood. CoA has a poor prognosis when untreated in childhood, due to complications such as aneurysm formation, aortic dissection, coronary artery disease, and intracranial hemorrhage. These complications result from arterial hypertension secondary to the coarctation and most patients die before reaching the age of 50 years from coronary heart disease, stroke, or sudden death. 1 2

All therapeutic options such as open surgery, balloon dilation, and stent implantation are effective in the treatment of CoA in adults. 3 The implantation of bare stents has become an excellent alternative to open surgery and balloon angioplasty, with better results. 4 However, bare stents are associated with notable complications, including aortic rupture, dissection, aneurysm formation, and even death. 5 In such complicated patients, implanting a covered stent is commonly used as a rescue treatment. Covered stents are widely used for the treatment of atherosclerotic abdominal and thoracic aneurysms in adults. 6 There is limited data in the literature about the use of covered stents in patients with aortic coarctation 7 with only few studies comparing covered with bare stents.

The aim of our study was to evaluate the hemodynamic effects of endovascular treatment for CoA by using invasive aortic catheterization. The short and intermediate-term outcomes with the use of bare Cheatham-Platinum (bCP) versus covered Cheatham-Platinum stents (cCP) (NuMed, Hopkinton, New York) are also presented.

## Materials and Methods

We included all patients with native CoA with post-balloon or port-surgical recoarctation who underwent treatment with bCP or cCP stent implantation between September 1, 2003, and February 1, 2019 at the “Onassis Cardiac Surgery Center,” in Athens, Greece. CoA was defined as the presence of systemic hypertension, with an upper-to lower-limb systolic blood pressure (BP) gradient of 20 mm Hg or more, confirmed by echocardiography, computed tomography (CT) angiography, or aortography. We also enrolled patients with native coarctation in addition to those with recoarctation after balloon angioplasty or any other surgical repair at the isthmus. At clinical presentation resistant hypertension was the indication for further investigation. Patients were treated with either the bCP or cCP stent implantation. The CP stent is made from an alloy of 90% platinum and 10% iridium. The cCP stent uses a gold soldering process and is fitted with a covering of expanded polytetrafluoroethylene and can be stretched up to a diameter of 12 to 24 mm

### Study Protocol and Endovascular Procedure

All patients underwent stenting for native CoA and recoarctation. Stent criteria selection has been published in previous reports. 5 8 9 The cCP stent was used in high-risk patients (age >40 years), tortuous aortic arch and isthmus, associated patent ductus arteriosus, near atresia, preexisting wall injury, reintervention and patients with Turner syndrome. The bCP stent was used in post-surgical recoarctation, when the lesion was close to the subclavian artery and according to the physician's preference. The procedures were performed under fluoroscopic guidance in the catheterization laboratory.

Patients were given antibiotic prophylaxis. After right femoral artery access was obtained, heparin sulfate (100 IU/kg body weight) was given intravenously and a 5F sheath was inserted. The stenotic lesion was crossed in a retrograde manner using a 5F right coronary Judkins catheter (Cordis Corporation, Miami, Florida) with the aid of a flexible-tip guidewire, 0.035 inches in diameter. Once the coarctation was crossed, BP was recorded to calculate the peak-to-peak systolic gradient. Aortography was then performed to optimize the anatomy and capture aortic dimensions. Angiography was performed in left lateral oblique and anteroposterior projections. Vascular closure devices were placed before dilatation because of the use of large introducing sheaths to avoid bleeding. The first wire was exchanged for an Amplatz super stiff, 0.035-inch, 260-cm wire (Cook Cardiology, Bloomington, Indiana), which was left in the right brachial artery, enabling the straightest course for subsequent stent deployment. Pre-dilation with a small balloon was strongly prohibited. The choice of stent diameter and length was important. Stent diameter was selected based on the diameter of proximal aorta or 1 to 2 mm larger. The stent should not exceed the diameter of the descending aorta in the level of the diaphragm. We aimed to cover the coarcted segment without occlusion of the subclavian artery.

The bCP or cCP stents were used in all procedures. The CP stent was hand-crimped down onto a balloon-in-balloon catheter (NuMed), which allowed a precise and safe stent delivery. A 12F long sheath for bCP stent and 14F sheath for cCP stent delivery was used. The diameter of the balloon was selected based on the proximal isthmus at the level of takeoff of the left subclavian artery. The length of the chosen stent was based on the distance between the left subclavian artery and 15 mm beyond the site of the CoA. After proper balloon size selection, rapid ventricular pacing at a rate 140 to 180 beats/min was instituted to reduce stroke volume, while maintaining reasonable BP. Immediate full dilation of the stent was performed, without exceeding the maximal pressure recommended by the manufacturer, which is 3 atm for 24 mm stent up to 7 atm for 12 mm stent. Hemodynamic data and angiographic measurements of the coarctation segment and other profiles were measured. A successful outcome was defined as a peak systolic pressure gradient after stent implantation of <15 mm Hg at the site of coarctation with no evidence of stent migration or aortic dissection. The implantation technique for covered stents was similar to bare stents, but required 2 to 3 Fr larger sheaths. On discharge, all patients received antiplatelet therapy for up to 6 months (aspirin 100 mg/d).

Patients were followed at 1, 3, 6, and 12 months after stent implantation and yearly thereafter. During the follow-up, patients underwent physical examination, BP measurements, and echocardiography. The chest X-ray, which was done on every follow-up, was useful in detecting stent migration. A CT was scheduled after 6 months to evaluate possible intimal hyperplasia, stent position, diameter at the dilated site, and the presence of an aneurysm (defined as >3 mm bulging).

We recorded all demographic and baseline patients' characteristics. Primary end points were aortic wall injury, aortic dissection, migration of stent during procedure, and vascular injuries. Recoarctation after the procedure was defined on the basis of a combination of clinical signs (arm-leg BP difference >20 mm Hg) and noninvasive imaging by echocardiography (BP gradient >20 mm Hg) or invasive gradient measurements by catheterization and >10% of the stent lumen obstruction due to intimal proliferation within the stent. Secondary end points included aneurysm and/or pseudoaneurysm formation, obstruction of left subclavian artery, hypertension, and need for antihypertensive therapy during follow-up period, or mortality.

### Statistical Analysis

Values were expressed as percentages (%) for categorical variables and mean with standard deviation for continuous variables. Numerical values were compared by independent sample *t* -test. Categorical parameters were compared by Chi-square test. To compare the mean values of the same patients before and after the procedure we used paired samples *t* -tests. A *p* -value <0.05 was considered significant.

## Results

A total of 47 patients, comprised of 17 adolescents (<18 years old) and 30 adults with a mean age of 28.59 ± 15.16 years, (range 11–65 years), mean weight of 69.59 ± 18.11 kg, mean height of 167.58 ± 11.45 cm, and mean body surface area 1.77 ± 0.25 m ^2^ participated in the study. Among them, 21 (44.7%) were males. The mean BP in the arm before intervention was 184.23 ± 23.95 mm Hg. No patients were presented with renovascular hypertension, while two patients had lower extremity fatigue on exercise or in long walking distance but not in terms of short-distance intermittent claudication. A total of eight patients had previous surgical repair for coarctation and nine patients had a previous balloon angioplasty with a total of 12 interventions. There was also a patient with Turner syndrome. A total of 48 CP stents were implanted: 24 bCp and 24 cCP. Patients in both groups did not differ in terms of demographic characteristics, type of previous CoA repair, other comorbidities or associated cardiac findings, including patent ductus arteriosus, bicuspid aortic valve, coronary artery disease, and aortic valve replacement. The technical success of the endovascular procedure was 100%. All patients reported improvement of their presenting signs with no recurrent symptoms.

### Results from Catheterization before and after Stenting

**Fig. 1:**
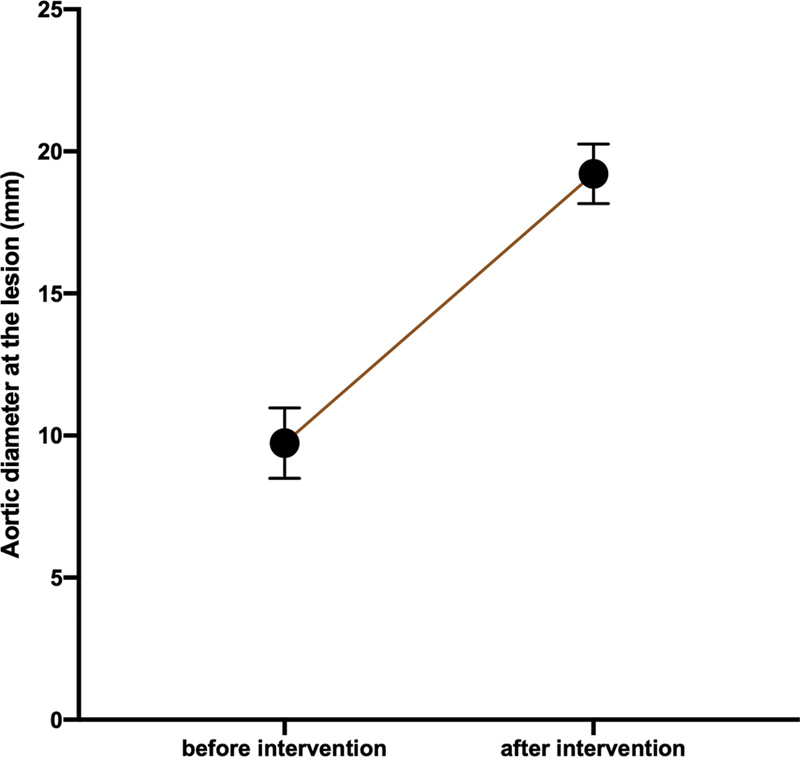
Error plot with 95% confidence intervals illustrating the mean aortic diameter (mm) at the coarctation lesion (before intervention = 9.7 ± 3.3 vs. after intervention = 19.2 ± 2.9; *p* <0.01).

**Fig. 2:**
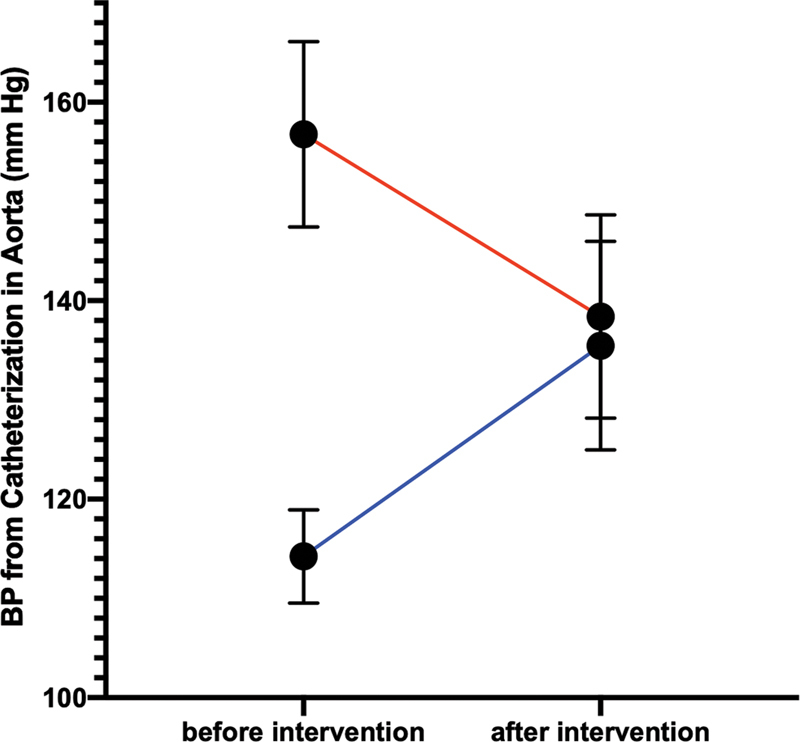
Error plots with 95% confidence intervals illustrating the mean blood pressure (BP; mm Hg) from catheterization both in the descending aorta (before intervention = 114.2 ± 12.8 vs. after intervention = 135.5 ± 28.1; *p* <0.01; *blue line* ) and in the ascending aorta (before intervention = 156.8 ± 25.0 vs. after intervention = 138.4 ± 27.5; *p* <0.01; *red line* ).

**Fig. 3:**
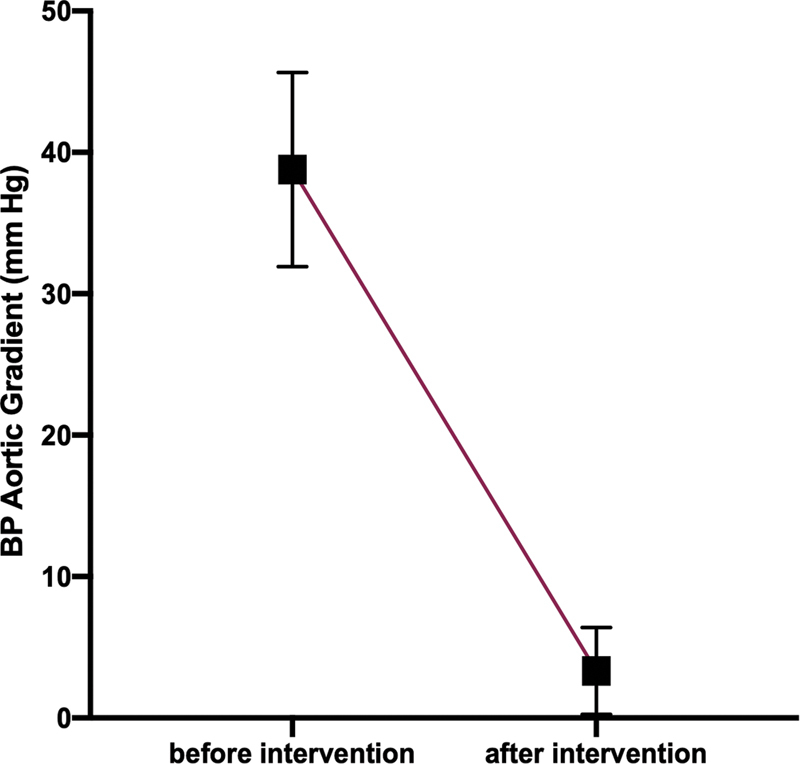
Error plots with 95% confidence intervals illustrating the mean difference in aortic blood pressure (BP) gradient (mm Hg; BP in ascending minus BP in descending aorta) from catheterization (before intervention = 38.8 ± 20.9 vs. after intervention = 3.3 ± 9.5); *p* <0.01.

The mean aortic diameter (mm) at the CoA lesion increased from 9.7 ± 3.3 to 19.2 ± 2.9 mm ( *p* <0.01) after the endovascular procedure ( Fig. 1 ). Hypertension was the indication for further investigation. The invasive mean BP (mm Hg) from catheterization in the descending aorta was increased after the endovascular intervention (before = 114.2 ± 12.8 vs. after = 135.5 ± 28.1; *p* <0.01), while the invasive mean BP (mm Hg) from catheterization in the ascending aorta was decreased after the endovascular intervention (before = 156.8 ± 25.0 vs. after = 138.4 ± 27.5; *p* <0.01; Fig. 2 ). The mean difference in invasive aortic BP gradient (mm Hg) (BP in ascending minus BP in descending aorta) was statistically significant after the endovascular intervention (before = 38.8 ± 20.9 vs. after = 3.30 ± 9.5; *p* <0.01; Fig. 3 ).

### Differences in Catheterization Results between Bare and Covered Stent

**Fig. 4:**
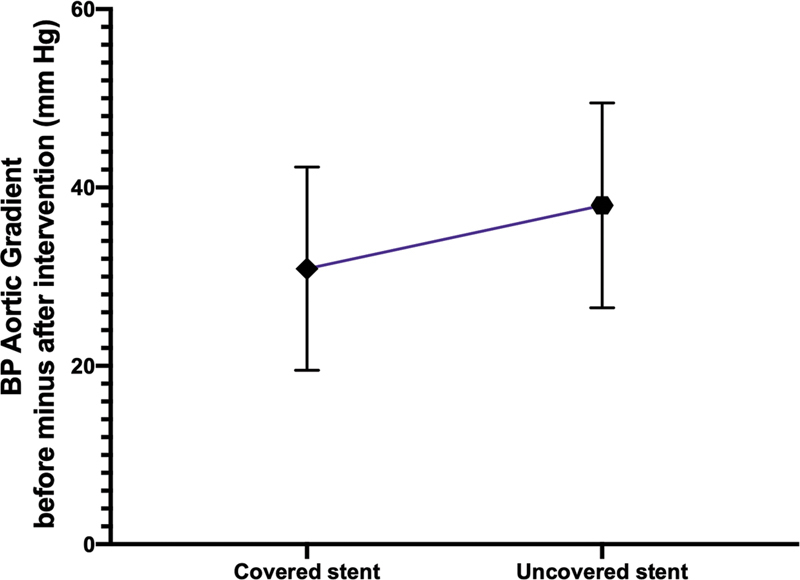
Error plot with 95% confidence intervals illustrating the mean decrease of aortic blood pressure (BP) gradient after intervention (BP aortic gradient before minus after intervention) for patients with covered (30.9 ± 23.6) and uncovered stents (38.0 ± 23.1) ( *p* = 0.36; not significant).

There was a reduction in the invasive aortic BP gradient (mm Hg) from 41.71 ± 18.64 to 2.06 ± 5.7 ( *p* <0.001) for cCP and from 45.46 ± 20.21 to 3.80 ± 13.19 ( *p* <0.001) for bCP stents. An increase in diameter (mm) from 9.88 ± 3.15 to 18.94 ± 3.09 ( *p* <0.001) for cCP and from 9.39 ± 3.14 to 19.43 ± 2.97 ( *p* <0.001) for bCP stents was also recorded. The mean decrease of aortic BP gradient after intervention (BP aortic gradient before minus after intervention) among patients with cCP (BP gradient = 30.9 ± 23.6 mm Hg) and bCP stents (BP gradient = 38.0 ± 23.1 mm Hg; *p* = 0.36; Fig. 4 ) did not reach levels of statistical significance.

### Complications and Other Secondary Outcomes during Follow-up

**Fig. 5:**
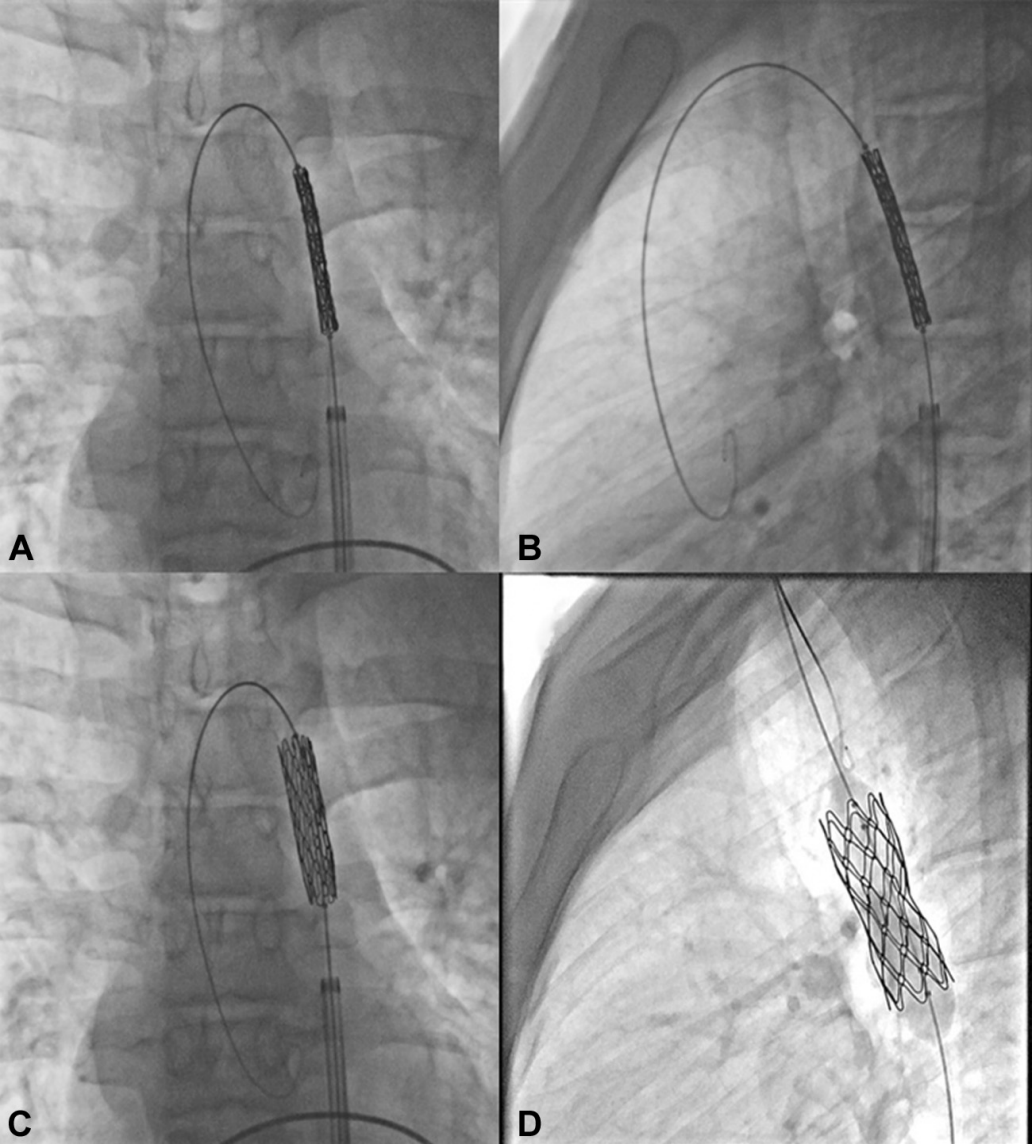
Steps of endovascular stent implantation for aortic coarctation; ( **A,B** ): positioning and ( **C,D** ) stent deployment.

**Fig. 6:**
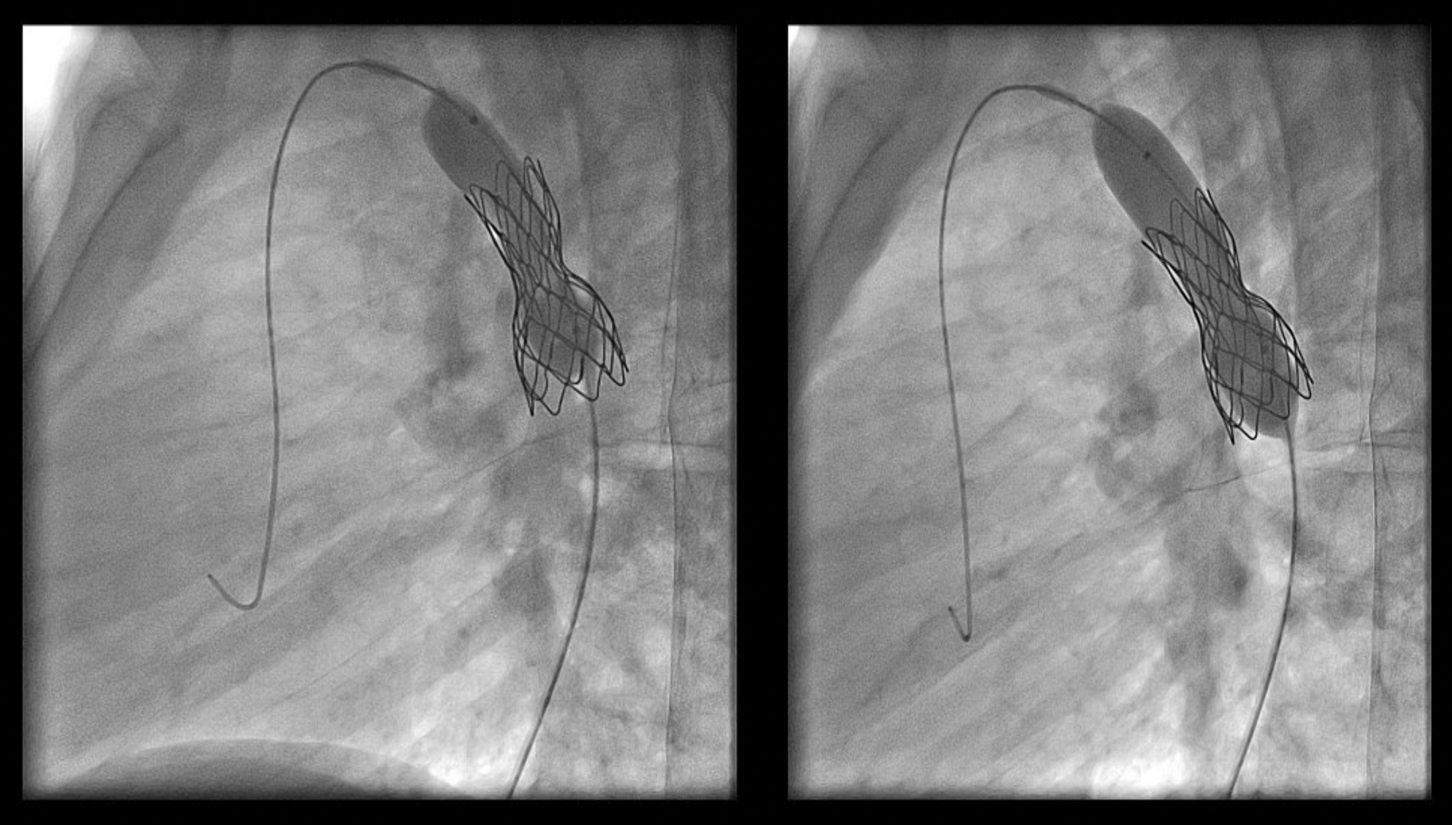
Ballooning in a patient who developed stent restenosis after previous coarctation stenting.

**Fig. 7:**
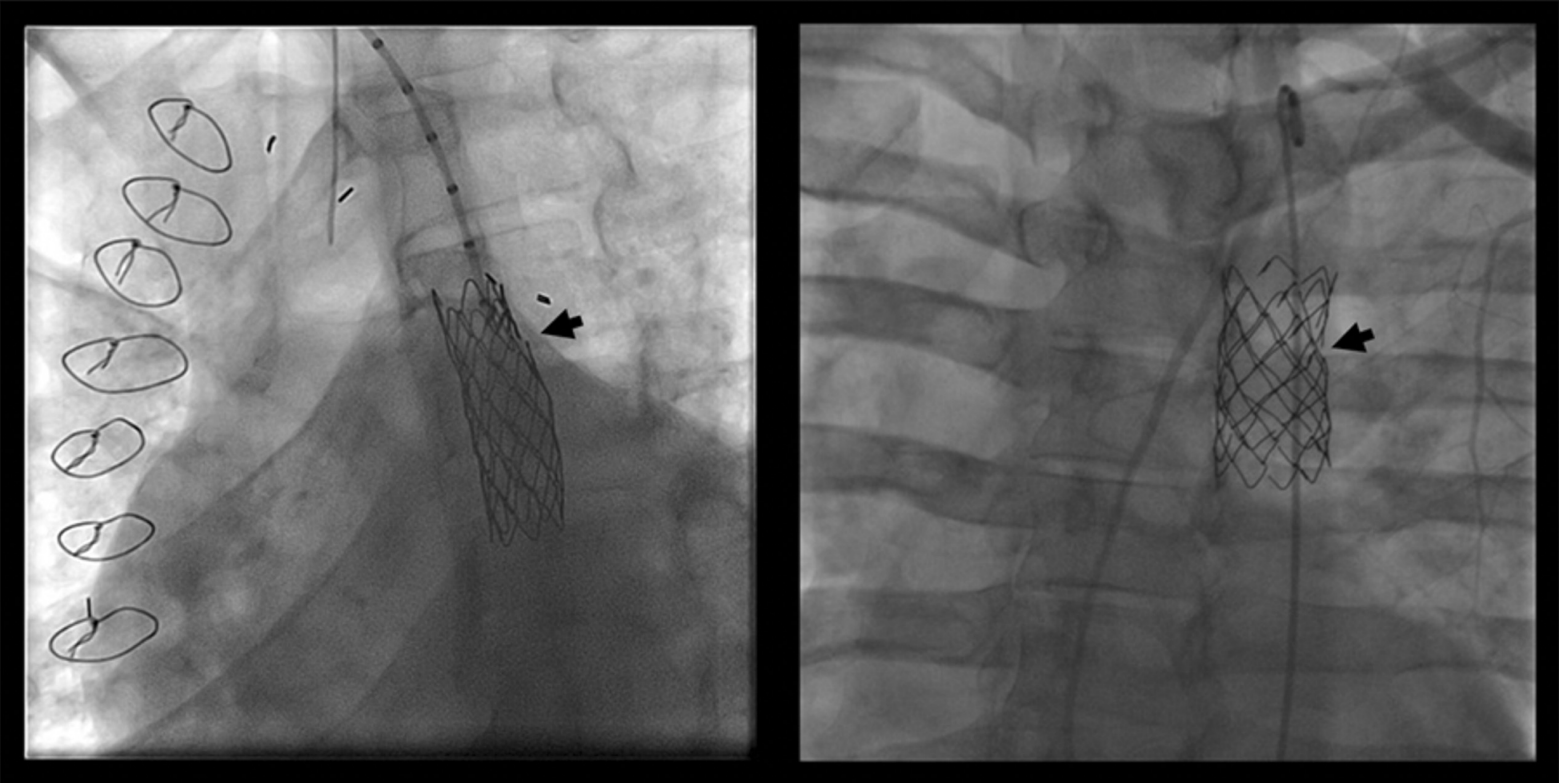
Stent fracture.

Steps of endovascular stent implantation for aortic coarctation were shown in Fig. 5 . Occurrence of recoarctation /restenosis with need for reintervention was seen in three patients (6.4%) from both groups: two in the cCP (4.34%) group, and one in bCP (2.17%) during follow-up. The recoarctation/restenosis cases included two males and one female patient. Recoarctation occurred 22, 60, and 74 months after stent implantation and was treated with re-dilatation ( Fig. 6 ); no recurrence or complications during the rest of follow-up was recorded. We accidentally recorded two stent fractures (4.24%) on bCP stent group with no clinical significance ( Fig. 7 ). There was also a stent migration to the descending aorta from the bCP stent group which was treated with stent re-dilatation and fixation with a new stent implantation. There was no incidence of paraplegia, bleeding, or vascular complications in our study cohort. No death, dissection, or pseudoaneurysm formation was discovered during follow-up. There was no statistical difference between the two groups of stenting concerning the duration of hospital stay (2.5 ± 2.6 days in the bCP vs. 1.6 ± 0.6 days in the cCP stent group). All patients were alive at a mean follow-up of 11.4 ± 4.1 years. Echocardiography follow-up was obtained in 33 patients. Maximal peak systolic velocity was decreased after the stent implantation from 3.5 ± 0.7 to 2.3 ± 0.8 m/s ( *p* <0.01).

## Discussion

Our study indicates that endovascular repair for CoA is a highly effective procedure, with minimal post-procedural complications. A significant increase in the mean aortic diameter at the CoA lesion was recorded after the procedure, which was also translated in a remarkable hemodynamic improvement in invasive aortic BP measurements: increase in the descending aorta and decrease in the ascending aorta BP. Notably, we recorded a trend toward higher mean decrease of aortic BP gradient and an increased aortic diameter after intervention among patients with uncovered, compared with patients with covered stents, although this did not reach statistical significance levels. A retrospective analysis of procedural data from 17 institutions showed successful stent placement without a significant residual gradient or serious complication in 97.9% of patients. 10

### Complications after Stent Implantation for Aortic Coarctation

Aortic wall complications such as dissection or aneurysm formation were not seen in our patients both for bare and covered stent group. Forbes et al 11 12 reported that aortic wall complications consisting of dissection or aneurysm formation were seen in 1% of the 302 total cases. The outcomes from the comparison of surgical treatment, balloon angioplasty alone, and endovascular stent placement in patients treated for coarctation using the Registry for the Congenital Cardiovascular Interventional Study Consortium (CCISC), 11 showed that stent implantation had significantly lower complication rates. Similarly, the Coarctation of the Aorta Stent Trial (COAST) in 2007, which was designed to assess the safety and efficacy of CP stent for the treatment of coarctation showed that all but one implantation was successfully completed. 13 In our group of bCP stent patients, we recorded two stent fractures; however, no hemodynamic effect was observed, and the patients are still under close observation. Stent fracture is common, but has been clinically insignificant. In the COAST trial, 23 fractured stents were identified, though none led to decreased stent integrity, stent migration, aortic wall injury, or hemodynamic obstruction. According to the data of Meadows et al 13 CP stent fractures were noted in two patients after 1 year, and in 11 patients after 2 years (among 104 treated), with evidence of fracture progression. However, stent fracture and progression of fracture did not result in clinically important sequelae.

### Comparing bCP with cCP Stents

Our study showed encouraging results with cCP stents for prevention of recoarctation in patients undergoing endovascular treatment of native CoA. Although recoarctation occurred in both groups (2.17 vs. 4.34%), the difference between groups regarding this complication was statistically nonsignificant. Most likely, increasing the sample size of this study would have resulted in a significant difference between groups on the occurrence of recoarctation. Theoretically the cCP stent can protect the post-stenotic area redirecting the flow away from the dilated areas. Additional structural support creates a protective barrier at the site of stent placement All these factors theoretically reduce the risk of acute vascular trauma as well as longer term aneurysm formation. When aortic aneurysm or stent fracture occurs with bCP stent placement, cCP stents are often used as a rescue therapy. The cCP stents could also be the initial transcatheter intervention of choice, especially in the setting of complex anatomy of the coarctation or in older patients with more friable and calcified aortic wall tissue. 14 However, cCP stents require larger sheath sizes, which limits their use in small children. Additionally, care must be taken to avoid occlusion of significant aortic branches, including paraspinal branches of the descending aorta, which can be difficult to identify. 15 In our series, no significant differences among patients with cCP and bCP stents were recorded. We treated one complication (stent migration/embolization) in the bCP stent group, which was managed with stent re-dilatation and fixation and one case of stent malposition (sliding) in the cCP stent group, which was successfully managed immediately in the same session with the implantation of a second covered stent.

### Follow-up after Stenting for Aortic Coarctation

Patients with coarctation should be followed throughout lifetime. For those who have undergone repair, this follow-up should be at least annually, with specific attention paid to baseline or exercise-induced hypertension. 16 BP control can be improved in adult patients after relieving the stenosis of coarctation. 17 We showed that the systolic gradient across the CoA and mean systolic and diastolic BP were reduced after both types of CP stent implantation. Our results are in accordance with others 17 18 that implantation of the cCP stent is associated with significantly improved hemodynamic status. In our study, normotensive cases increased after procedure in 77% of cases. These findings are indicative of the effectiveness of stent on improvement of patient's hemodynamics. Hypertension is endemic in patients with aortic coarctation, even if no residual coarctation exists, and it must be appropriately treated. 19 Additionally, imaging of the repaired coarctation should be performed at least every 5 years, or sooner based on original anatomy and symptoms, to assess the coarctation repair site for complications like aortic aneurysm or recurrent stenosis. 16

### Long-Term Results

For the evaluation of late complications, such as aneurysm formation and recoarctation, patients should be followed by magnetic resonance angiography (MRA) or CT angiography. 20 During the follow-up period, recoarctation occurred only in the bCP group. Use of cCP stents for complex and very severe coarctations, especially in older patients with relatively less compliant aortic walls, has been associated with encouraging results. 17 21

### The Effect of Stenting in BP Control

Hypertension was the indication for further investigation. The BP control can be improved in adult patients after relieving the stenosis of coarctation. 17 We showed that the systolic gradient across the CoA and mean systolic and diastolic BP were reduced after both types of CP stent implantation. Our results are in accordance with others 17 18 that implantation of the cCP stent is associated with significantly improved hemodynamic status. In our study, normotensive cases increased after procedure in 77% of cases. These findings are indicative of the effectiveness of cCP stent on improvement of patient's hemodynamics. In other studies, reduction or discontinuation of anti-hypertensive medication following stent implantation was achieved in 18 to 88% of the patients. 15 22 Overall, in our study, antihypertensive therapy was reduced but not discontinued in majority of patients. To our practice, we prefer lifetime anti-hypertensive medication because hypertension is endemic in patients with aortic coarctation, even if no residual coarctation exists, and it must be appropriately treated. 15 19 However, the improvement in hypertension control was not different between groups. It is notable that there is no report of any procedure-related mortality in cCP or bCP stenting in the literature, whereas it is reported that death may occur in 0 to 1.4% of cases after non-CP bare stenting. 5 Patients were discharged at a mean of 2 days after the procedure with no difference between groups. In our study there was no death.

## Conclusion

Endovascular stenting is efficient in treating patients with CoA. The bCP or cCP stents, are a safe treatment option with minimal post-procedural complications.
